# Evaluation of sunobinop for next-day residual effects in healthy participants

**DOI:** 10.3389/fphar.2024.1432902

**Published:** 2024-08-19

**Authors:** Alessandra Cipriano, Ram P. Kapil, Mingyan Zhou, Manjunath S. Shet, Stephen C. Harris, Glen Apseloff, Garth T. Whiteside

**Affiliations:** ^1^ Imbrium Therapeutics, Stamford, CT, United States; ^2^ Ohio Clinical Trials, Inc., Columbus, OH, United States

**Keywords:** sunobinop, insomnia, next-day residual effects, healthy participants, nociceptin/orphanin FQ

## Abstract

Sunobinop is a novel, potent, selective partial agonist at nociceptin/orphanin FQ peptide (NOP) receptors. The primary objective of this randomized, double-blind, placebo-controlled study was to assess the next-day residual effects of an evening dose of sunobinop in healthy participants. Participants were randomized into 1 of 5 treatment sequences. Treatment consisted of 1 dose each of sunobinop 0.2, 0.6, 2, and 6 mg suspension and placebo suspension. Key pharmacodynamic (PD) measures included the digit symbol substitution test (DSST), Karolinska sleepiness scale (KSS), and body sway. The randomized safety population consisted of 25 participants. The DSST, KSS, and body sway showed dose-dependent effects following the administration of sunobinop, with no significant differences versus placebo at sunobinop doses <2 mg. At sunobinop 2 mg, PD effects were relatively small in magnitude and inconsistent. The last timepoint where significant differences between sunobinop 2 mg and placebo on the DSST, KSS, and body sway were observed was at 12 h, 16.5 h, and 13.5 h postdose, respectively. Sunobinop 6 mg resulted in larger and consistent PD effects, with significant differences from placebo at all timepoints up to 16.5–18 h postdose. Somnolence was the most frequently reported adverse event (AE), and all AEs were mild-to-moderate. No deaths occurred during the study or discontinuations due to an AE. Overall, a nighttime oral dose of sunobinop up to 2 mg was safe and generally well tolerated in healthy participants with limited next-day residual effects that were consistent with other sedative/hypnotic drugs.

## Introduction

Sunobinop is a potent, selective, partial agonist at nociceptin/orphanin FQ peptide (NOP) receptors (Whiteside et al., 2024). It does not activate mu and kappa opioid receptors and is a low-affinity, weak, partial agonist at delta opioid receptors (Whiteside et al., 2024).

Sunobinop may have therapeutic utility in the treatment of insomnia. In rats, sunobinop decreased wakefulness and increased non-REM sleep, with effects nearly abolished in NOP knockout rats, confirming these effects were mediated via activation of NOP (Whiteside et al., 2024). Importantly, no significant effects on learning, memory, reward, respiration, or intestinal transit were observed at doses substantially higher than required for therapeutic effect.

Sunobinop has been studied in 3 phase 1 pharmacokinetic studies (Cipriano et al., 2024). In healthy subjects, sunobinop demonstrated rapid absorption across a 3- to 30-mg dose range, with a half-life of 2.1–3.2 h, indicating once-daily dosing is appropriate (Whiteside et al., 2024). Results of these studies found that sunobinop was rapidly eliminated unchanged via urine, suggesting exclusive renal elimination without hepatic metabolism. Dose-limiting absorption was observed above 10 mg (Whiteside et al., 2024). Sunobinop was safe and generally well-tolerated, with sedation/somnolence occurring as the most common treatment-related adverse event (AE). Across these 3 studies, 1 used daytime dosing, 1 used nighttime dosing, and the third utilized a crossover design. Based on the results of these studies, it was determined that the PK profile of sunobinop was similar between daytime and nighttime administration and resulted in comparable rates of sedation (Cipriano et al., 2024).

In patients with insomnia, a 10 mg dose administered at nighttime significantly improved sleep efficiency (the primary endpoint), reduced sleep latency, decreased wake time after sleep onset, and reduced nighttime awakenings (Whiteside et al., 2024). Sunobinop also altered sleep stage distribution and improved perceived sleep quality. Sunobinop was generally well-tolerated in both healthy subjects and patients, with no deaths, serious adverse events, or discontinuations due to AE; however, next-day residual effects were observed. Common side effects included fatigue/somnolence, euphoria, and dizziness. No clinically relevant changes were observed in laboratory results, electrocardiograms (ECGs), or oxygen saturation. The next-day residual effects observed with the 10 mg dose indicate a need for dose-ranging studies to elucidate these effects better; however, taken together, these results show that sunobinop demonstrates potential as a novel insomnia treatment, with NOP activation representing an attractive new treatment approach.

In addition to insomnia, sunobinop has potential therapeutic utility in an array of clinical disorders in which sleep disruption co-occurs and nighttime dosing would be desirable, including alcohol use disorder (AUD), insomnia in patients with AUD, overactive bladder, and interstitial cystitis/bladder pain syndrome (Lambert, 2008; Witkin et al., 2014; Anand et al., 2016; Whiteside et al., 2023; Whiteside et al., 2024). Because sunobinop is a centrally active compound intended to be administered at bedtime and next-day residual effects have been observed at 10 mg, this study aimed to evaluate the next-day residual effects of sunobinop across a range of doses.

## Methods

### Study design

**FIGURE 1:**
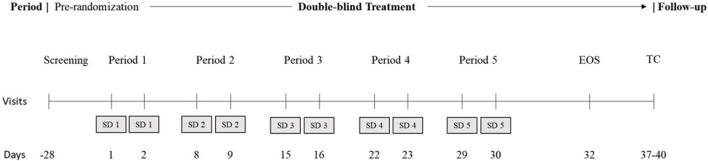
Study design. EOS, end of study; SD, study drug; TC, telephone call. During the 5 treatment periods, all participants were administered a placebo on day 1 for acclimatization to the overnight laboratory environment and active treatment on day 2, per the randomized schedule. There was a minimum washout of approximately 5 days between successive treatment periods. Subjects were confined to the study unit from check-in of each period to the morning of day 4 in each period. EOS procedures were performed before discharge for all participants, including those who discontinued the study. A follow-up telephone call was conducted after EOS or after early withdrawal. The total study duration was up to approximately 64 days.

This was a single-center, randomized, double-blind, placebo-controlled, 5-period, crossover study in healthy adult male and female participants. Figure 1 shows the study design diagram. Briefly, the study consisted of screening and pre-randomization, 5 double-blinded treatment periods to the end of the study, and then a follow-up telephone call.

An institutional review board reviewed and approved the protocol, administrative change documents, amendment, and informed consent forms before participants were screened. All participants provided informed consent before any protocol-specific procedures.

### Inclusion and exclusion criteria

The study inclusion criteria consisted of males and females aged 18–50 years (inclusive), a body weight of 50–100 kg and a BMI of 18–30 kg/m^2^, no psychiatric history within 5 years of screening, and healthy and free of significant abnormal findings as assessed by medical history, physical examination, clinical labs, vital signs, and ECG.

Participants were excluded from the study if they were pregnant or lactating, had a history (within 5 years) of drug or alcohol abuse, were a night or rotating shift worker, or had any history of seizures or head trauma with sequelae.

### Screening and check-in

A screening visit was conducted within 28 days before check-in. The information obtained from screening included informed consent, medical and medication history, demographic data, physical exam, inclusion/exclusion criteria response, vital signs (systolic/diastolic blood pressure, pulse rate, and respiratory rate), pulse oximetry (SpO2), oral temperature, ECG, and alcohol evaluation.

Laboratory assessments were conducted, including clinical labs, serum pregnancy tests for female participants, serum follicle-stimulating hormone in post-menopausal females only, and screens for alcohol, cotinine, drugs of abuse, hepatitis B, and hepatitis C.

Participants were confined to the clinical site from check-in of each period to the morning of day 4 in each study period. During check-in, clinical labs, drug/alcohol/cotinine screening, and pregnancy screening were conducted. Additionally, vital signs, SpO2, and oral temperature were measured, and an ECG was obtained.

### Drug treatment

Participants were randomized into 1 of the 5 treatment sequences, each receiving all treatments according to their randomly assigned sequence. In each period, participants received 2 consecutive, double-blind study drug administrations (active/placebo or placebo/placebo). Aqueous suspensions offer more flexibility while studying a range of doses, and previous studies of sunobinop have shown similar PK profiles for aqueous suspension, tablet, and sublingual dosing (Cipriano et al., 2024). Thus, a 20-mL aqueous suspension of sunobinop 0.2, 0.6, 2, and 6 mg (in 0.5% methyl cellulose in water) and placebo (consisting of the same vehicle in the same volume and an identical administration procedure to active) were used. Following initial suspension administration, 4 successive 20-mL rinses with water were consumed to ensure complete dose administration. Participants then consumed sufficient water to bring the total volume to 240 mL. Each participant was dosed in the evening at bedtime at least 2 h after fasting and 30 min before lights out. After unblinding, it was revealed to the investigators that all participants received placebo on night 1 and, except for the placebo sequence, active treatment on night 2. Night 1 placebo dosing was intended for acclimatization to the overnight laboratory environment. There was a minimum washout of approximately 5 days between successive treatment periods.

### End of study or early discontinuation and follow-up

At the end of the study or early discontinuation, all participants had completed clinical lab evaluations and were screened for pregnancy. Additionally, vital signs, SpO2, and oral temperature were measured, and an ECG was obtained for all participants. A follow-up call was made to each subject 7–10 days after the last study drug administration to assess AEs.

### Endpoints

The primary objective of this study was to assess the next-day residual effects of an evening dose of sunobinop on pharmacodynamics (PD) as measured by the digit symbol substitution test (DSST), (Wechsler, 1981), Karolinska sleepiness scale (KSS), (Akerstedt and Gillberg, 1990), and body sway test. Secondary endpoints included AEs assessed by nonleading inquiry, vital signs (systolic and diastolic blood pressure, heart rate, respiratory rate, temperature), SpO2, ECG, and physical examination.

### Pharmacodynamic and safety assessments

Following prior evening dosing, participants were administered PD assessments the next morning beginning 30 min after lights on to evaluate next-day residual effects at 8-, 9-, 10.5-, 12-, 13.5-, 15-, 16.5-, 18-, and 22-h postdose (corresponding to 0.5, 1.5, 3, 3.5, 6, 7.5, 9, 10.5, and 14.5 h after awakening). The DSST examines attention and psychomotor speed. (Jaeger, 2018). Participants were presented with a code table in which numbers (1–9) were matched with a symbol and asked to fill in squares below digits with the corresponding symbol. The outcome measure was the number of correctly completed pairs in 90 s, with higher scores reflecting better attention and psychomotor speed. The KSS measures sleepiness using a 9-point Likert scale ranging from “1 = extremely alert” to “9 = very sleepy–fighting sleep” (Akerstedt et al., 2014). Thus, higher scores on the KSS indicate greater sleepiness. A paper-and-pencil version of the KSS was administered approximately 30 min after lights on. The body sway test measures postural stability and was assessed using a custom swaymeter (Innovation Creation Ltd., Buckinghamshire, United Kingdom). Participants stood on a premarked mat with eyes closed and arms along the side of the body for 60 s. Sway (postural stability) was defined as the cumulative magnitude of all movements over 60 s. Higher scores on the body sway test indicate worse postural stability.

Clinical laboratory assessments included biochemical analysis, hematology, urinalysis, serology, screens for drugs of abuse, as well as pregnancy and follicle-stimulating hormone (self-reported postmenopausal females only) screens. Vital signs, SpO2, and oral temperature were measured at each treatment period. AEs and serious AEs were detected, documented, classified, and reported by the investigator or their designees throughout the study. All AEs were recorded from the time of informed consent until the end of the follow-up period, regardless of seriousness. All management or treatments required for AEs were recorded.

### Statistical methods

The randomized safety population consisted of all randomized participants who received at least 1 dose of the study drug, and the full analysis population consisted of all participants who also had at least one valid PD measure.

Continuous demographic and baseline variables were summarized using n, mean, and standard deviation. For categorical variables, the number and percentage of participants were calculated.

Descriptive statistics were tabulated by treatment for next-day residual effects. Comparisons were made predose on night 2 to each of the postdose timepoints the following day. The data from PM dosing on night 2 in the placebo period were used as placebo data.

Next-day residual effects were statistically analyzed using a mixed model for repeated measures approach, with fixed effects for period baseline, sequence, treatment period, and time point, the interaction of treatment by time, a random effect for participants nested within the sequence, and time as a repeated measure. Descriptive statistics were tabulated by treatment for AEs. AEs were coded to MedDRA (version 18.1) terms and summarized by treatment.

## Results

### Subject disposition

A total of 76 participants enrolled in the study, and 51 failed screening. The randomized safety population comprised 25 participants; 23 (92%) completed the study. Two participants discontinued the study due to personal choice.

### Baseline demographics and characteristics

Participants ranged in age from 24–48 years, with a mean age of 35.8 years. The overall mean body mass index was 26.85 kg/m^2^. Of the 25 randomized participants, 19 (76%) were male. Most participants were white (12 of 25 participants; 48%) or black or African American (12 of 25 participants; 48%).

### Next-day residual effects

#### DSST

**FIGURE 2:**
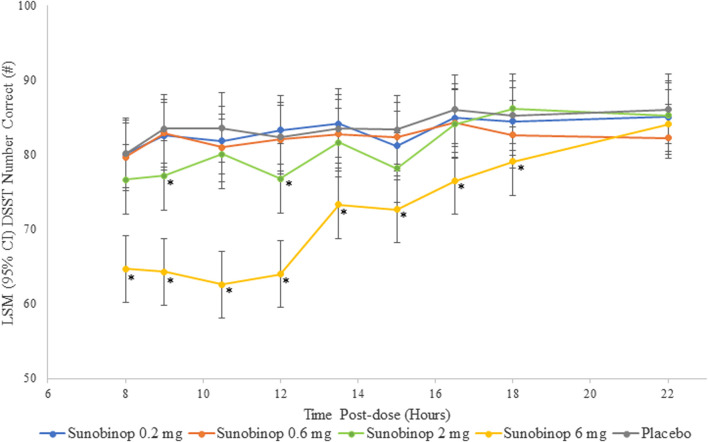
DSST scores vs. time by treatment. ^*^ *p* < .05 compared with placebo. CI, confidence interval; DSST, digit symbol substitution test; LSM, least squares mean. The DSST showed a dose-dependent decrease in correct responses with active treatment, indicating an effect on attention and psychomotor speed.

The DSST showed a dose-dependent decrease in correct responses with active treatment, as shown in Figure 2. There were no statistically significant differences in the number of correct responses after administering sunobinop 0.2 mg and sunobinop 0.6 mg compared with placebo at any time point after dosing.

Sunobinop 2 mg had a small and variable effect on reducing the number of correct responses, suggesting a degree of impairment from baseline in attention/psychomotor speed that was statistically significant compared with placebo at 9 (mean difference, −6.3; *p* = .021) and 12 (mean difference, −5.6; *p* = .039) hours after dosing but was not significantly different at 8 (mean difference, −3.5) or 10.5 (mean difference, −3.5) hours postdose. Sunobinop 6 mg had a consistent and larger effect on number of correct responses, with statistically significant differences compared with placebo at all time points from 8 (mean difference, −15.6; *p* < .001) to 18 (mean difference, −6.1; *p* = .021) hours after dosing.

#### KSS

**FIGURE 3:**
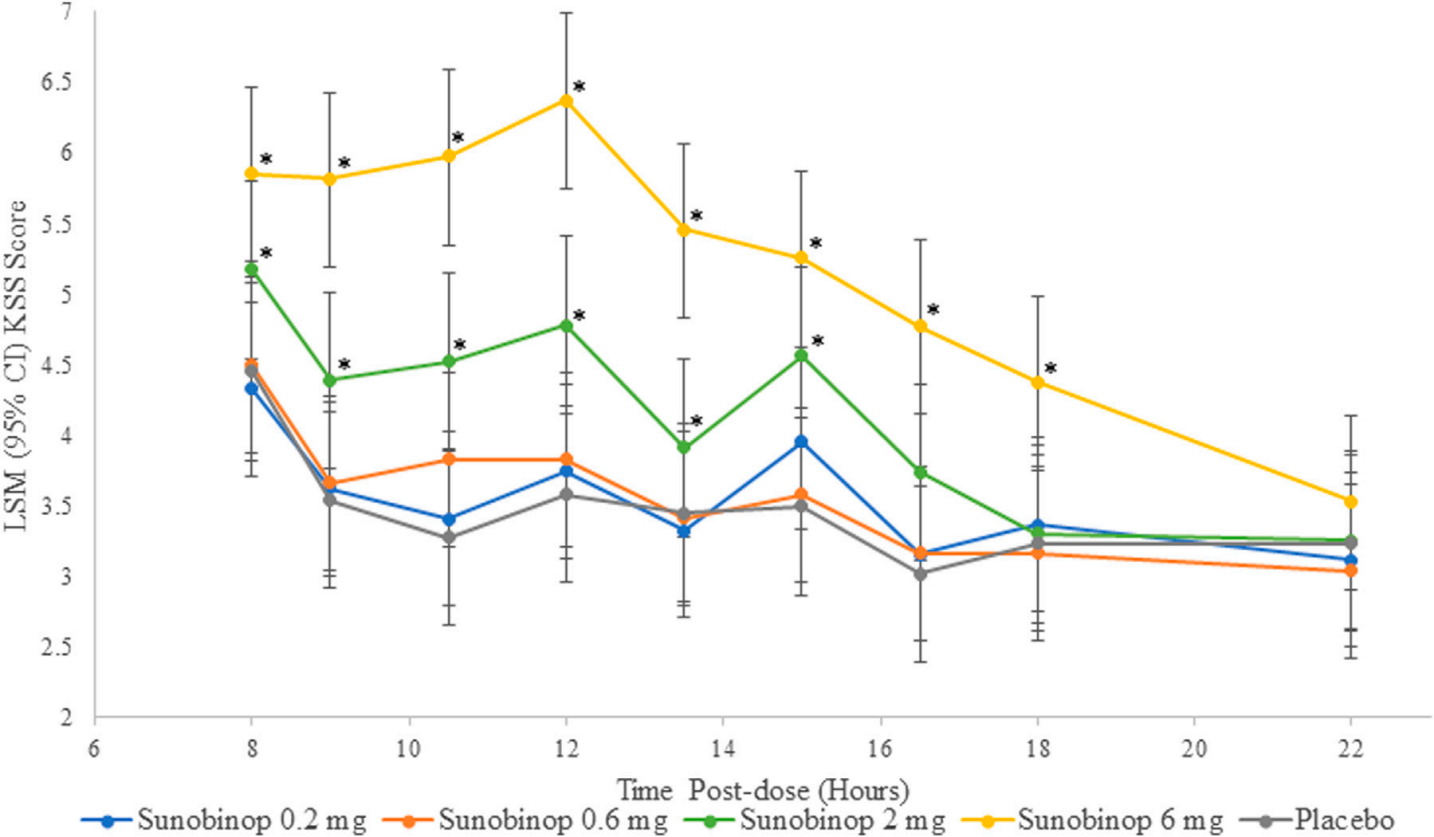
KSS vs. time by treatment. ^*^ *p* < .05 compared with placebo. CI, confidence interval; KSS, Karolinska sleepiness scale, LSM, least squares mean. The KSS scores showed a dose-dependent increase in feelings of sleepiness with active treatment.

The KSS scores showed a dose-dependent increase in feelings of sleepiness with active treatment, as shown in Figure 3. There were no statistically significant differences in KSS scores after administration of sunobinop 0.2 mg and 0.6 mg compared with placebo at any time point after dosing.

There were small but statistically significant differences in sleepiness as reflected by KSS scores after administration of sunobinop 2 mg compared with placebo at all time points from 8 (mean difference, 0.7; *p* = .042) to 16.5 (mean difference, 0.7; *p* = .042) hours after dosing except for the 13.5-h time point (mean difference, 0.5). There were larger statistically significant differences in KSS scores after administration of sunobinop 6 mg compared with placebo consistently at all time points from 8 (mean difference, 1.4; *p* < .001) to 18 (mean difference, 1.1; *p* = .001) hours after dosing.

#### Body sway test

**FIGURE 4:**
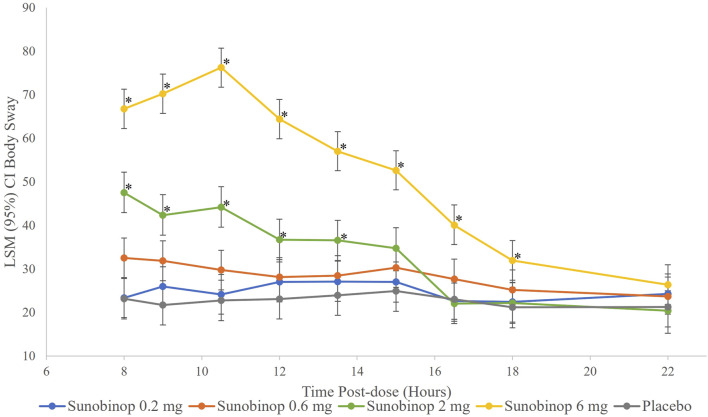
Body sway vs. time by treatment. ^*^ *p* < .05 compared with placebo. CI, confidence interval; LSM, least squares mean. The body sway test showed a dose-dependent increase in instability with active treatment, indicating worse postural stability.

The body sway test showed a dose-dependent increase in instability with active treatment, as shown in Figure 4, indicating worse postural stability. There were no statistically significant differences in body sway after administration of sunobinop 0.2 mg and sunobinop 0.6 mg compared with placebo at any time point after dosing.

There were small but statistically significant differences in body sway, indicating worse postural stability, after administration of sunobinop 2 mg compared with placebo at all time points from 8 (mean difference, 24.4; *p* < .001) to 13.5 (mean difference, 12.6; *p* = .044) hours after dosing. Sunobinop 6 mg had a larger and consistent effect on body sway that was statistically significant compared with placebo at all time points from 8 (mean difference, 43.6; *p* < .001) to 16.5 (mean difference, 17.0; *p* = .005) hours after dosing.

#### Safety

A summary of AEs is shown in Table 1. Overall, there were 158 AEs reported, and 24 of 25 (96%) participants experienced at least 1 AE (sunobinop 0.2 mg, 33%; sunobinop 0.6 mg, 46%; sunobinop 2 mg, 78%; sunobinop 6 mg, 92%). Somnolence was the most frequently reported AE, occurring in 88% of participants (sunobinop 0.2 mg, 12%; sunobinop 0.6 mg, 16%; sunobinop 2 mg, 61%; sunobinop 6 mg, 76%; placebo, 13%).

**TABLE 1:** summary of AEs.

System organ class^a^	0.2 mg (N = 24) n (%) R^b^	0.6 mg (N = 24) n (%) R^b^	2 mg (N = 23) n (%) R^b^	6 mg (N = 25) n (%) R^b^	Placebo (N = 23) n (%) R^b^	Overall (N = 25) n (%) R^b^
Any treatment-emergent AE	8 (33) 13	11 (46) 22	18 (78) 42	23 (92) 62	11 (48) 19	24 (96) 158
Mild Moderate Severe	6 (25) 11 2 (8) 2 0	8 (33) 18 3 (13) 4 0	7 (30) 26 11 (48) 16 0	6 (24) 33 17 (68) 29 0	5 (22) 9 6 (26) 10 0	4 (16) 97 20 (80) 61 0
Nervous system disorders						
Somnolence						
Mild Moderate	2 (8) 3 1 (4) 1	2 (8) 3 2 (8) 2	5 (22) 8 9 (39) 11	5 (20) 9 14 (56) 14	0 3 (13) 6	5 (20) 23 17 (68) 34
Headache						
Mild Moderate	3 (13) 3 0	2 (8) 3 0	4 (17) 4 1 (4) 1	4 (16) 7 1 (4) 1	4 (17) 4 0	8 (32) 21 2 (8) 2
Balance disorder						
Mild Moderate	0 1 (4) 1	1 (4) 1 0	1 (4) 2 2 (9) 2	1 (4) 1 4 (16) 4	0 0	3 (12) 4 5 (20) 7
Disturbance in attention						
Mild Moderate	0 0	1 (4) 1 0	0 0	1 (4) 1 1 (4) 1	0 0	2 (8) 2 1 (4) 1
Psychiatric disorders						
Abnormal dreams						
Mild	2 (8) 2	4 (17) 4	1 (4) 1	2 (8) 2	1 (4) 1	5 (20) 10
Insomnia						
Moderate	0	2 (8) 2	1 (4) 1	2 (8) 2	0	4 (16) 5
Irritability						
Mild Moderate	0 0	1 (4) 1 0	2 (9) 3 0	2 (8) 2 0	0 1 (4) 1	2 (8) 6 1 (4) 1
General disorders and administration site conditions						
Fatigue						
Mild Moderate	0 0	3 (13) 3 0	2 (9) 3 0	1 (4) 1 1 (4) 1	1 (4) 1 0	5 (20) 8 1 (4) 1

^a^ If a subject reported more than 1 occurrence of the same preferred term, the maximum severity was included in this table. Missing severities were treated as severe. Severity was taken from the severity the investigator reported on the electronic case report form.

^b^ R, number of reports.

AE, adverse event.

N, number of participants who received the specified treatment in the randomized safety population.

n, number of participants with at least 1 occurrence of an AE.

AEs reported by ≥ 2 participants for any treatment were somnolence, headache, balance disorder, disturbance in attention, abnormal dreams, insomnia, irritability, and fatigue. Most AEs (88%) were mild in severity. Except for 1 moderate AE (limb injury) and 1 mild AE (abnormal dreams), all AEs resolved by the end of the study. No significant changes in clinical lab values, vital signs, SpO2, or ECG results were observed. There was no evidence of drug-related crystalluria or hematuria. No deaths or serious AEs occurred during the study, and no subject discontinued due to an AE.

## Discussion

This study aimed to evaluate the next-day residual effects of an evening dose of sunobinop in healthy participants. A consistent dose-dependent trend following the administration of sunobinop was observed on all the PD endpoints, with no statistically significant differences between sunobinop 0.2 and 0.6 mg and placebo at any time point.

After administration of sunobinop 2 mg, analysis of DSST scores, assessing attention and psychomotor speed, showed a decrease in the number of correct responses, with statistically significant differences inconsistently observed up to 12 h compared to placebo. Similarly, after the administration of sunobinop 6 mg, the DSST also showed a decrease in the number of correct responses, with statistically significant differences consistently observed for up to 18 h compared to placebo. Analysis of KSS scores showed a dose-dependent increase in feelings of sleepiness following sunobinop 2 mg, which was statistically significant for up to 16.5 h compared with placebo. The LS mean was 5.2 (between neither alert nor sleepy and some signs of sleepiness) for sunobinop 2 mg and 4.5 (between neither alert nor sleepy and rather alert) for placebo (mean difference, 0.7) at 8 h postdose. The 8-h timepoint corresponds with the waking hour where normative data show average KSS scores of 4.5 in the AM, suggesting the effect of sunobinop 2 mg is minimal and well below the high-risk criterion of >8 for driving accidents (Akerstedt et al., 2017). For sunobinop 6 mg, the KSS showed an increase in feelings of sleepiness, which was statistically significant compared with placebo for up to 18 h. The LS mean was 5.8 (between neither alert nor sleepy and some signs of sleepiness) for sunobinop 6 mg and 4.5 (between rather alert and some signs of sleepiness) for placebo at 8 h. The body sway test also showed a dose-dependent increase in instability with active treatment, indicating worse postural stability. This test showed statistically significant differences up to 13.5 h after administration of sunobinop 2 mg and 16.5 h after administration of sunobinop 6 mg compared with placebo. The most frequently reported AE (88% of participants) was dose-dependent somnolence. All AEs were of mild-to-moderate severity. No deaths occurred during the study, and no reported discontinuations were due to an AE.

Sunobinop has also been studied in individuals with insomnia according to the Diagnostic and Statistical Manual of Mental Disorders, Fifth Edition (Association, 2013). Consistent with the results of this study, sunobinop doses of 0.5 mg, 1 mg, 3 mg, and 6 mg administered nightly for 2 nights showed dose-dependent next-day residual effects as assessed by the DSST. Sunobinop 1 mg showed no difference from placebo at 9 h, thus demonstrating that the therapeutic duration of effect wears off within the anticipated “sleeping” hours at this dose.

Other studies have investigated the next-day residual effects of sedative hypnotics. In 16 individuals with primary insomnia, trazodone, an antagonist of serotonin type 2 receptors and adrenoreceptors and an inhibitor of serotonin reuptake and a widely prescribed sleep aid, (Khouzam, 2017), was assessed in a 3-week, randomized, double-blind, placebo-controlled study. Trazodone 50 mg, the dose commonly prescribed for sedative/hypnotic purposes, was administered 30 min before bedtime for 7 days, and PD assessments approximately 8 h postdose on nights 1 and 7 for each treatment (active and placebo) resulted in significantly impaired short-term memory, verbal learning, body sway, and muscle arm endurance (Roth et al., 2011). While trazodone significantly differed from placebo in body sway at 8 h, it is unknown whether the next-day residual effects persisted beyond 8 h, making it difficult to compare the results to this study.

As in the current study, hypnotic agents, including zolpidem, suvorexant, and ramelteon, have been investigated in healthy participants. Zolpidem is a non-benzodiazepine GABAa positive allosteric modulator, suvorexant is an dual orexin antagonist, and ramelteon is a melatonin agonist (Bouchette et al., 2024). A single-blind pharmacokinetic assessment of therapeutic doses of zolpidem (5 mg), suvorexant (10 mg), and ramelteon (4 mg) was conducted in a randomized, active- and placebo-controlled trial in healthy 60–75-year-old Japanese participants for 4 weeks. The body sway test (measured at 14 h post-administration) found that all treatments produced significant body sway movements, with zolpidem resulting in a significantly smaller effect than suvorexant or ramelteon (Uemura et al., 2022). While not directly comparable, the next-day residual effects of these commonly used hypnotic agents were consistent with those of sunobinop 2 mg.

Similarly, in healthy men aged 18–45 years, suvorexant was evaluated in a randomized, double-blind, placebo-controlled trial. Next-day residual effects and psychomotor performance were evaluated at 10 h postdose. Suvorexant 100 mg (supratherapeutic dose) produced a statistically significant increase in reaction time for both simple reaction time and choice without a statistically significant effect on the DSST (Sun et al., 2013). Likewise, in another study, 10 mg zolpidem (therapeutic dose) and 0.25 mg triazolam (therapeutic dose) had short-lasting effects in healthy participants aged 20–31 years on memory, psychomotor performance, and postural sway at 1- and 4-h post-administration. There were no residual effects from zolpidem and triazolam at 6 and 8 h, respectively (Berlin et al., 1993). Conversely, in another study of healthy participants, cognitive impairment persisted up to 8.25 h postdose for participants who were administered triazolam 0.25 mg and zolpidem 20 mg (supratherapeutic dose) (Troy et al., 2000). Lastly, in another randomized, double-blind study, lemborexant 5 mg and 10 mg were compared with zolpidem tartrate extended-release 6.25 mg or placebo in healthy participants aged ≥55 years. When body sway was assessed in the morning, neither dose of lemborexant was associated with a significant change from baseline. However, body sway in the zolpidem group was significantly greater than placebo (Murphy et al., 2020).

Direct comparisons of hypnotic medications from different studies with differing methodologies are inappropriate due to differences in study design, including PD measures and time points assessed. For example, many studies only evaluate next-day residual effects up to 8–10 h postdose; thus, it is unclear whether these effects persist further into the awake hours. But overall, the results from the studies described above show that most hypnotic agents produce next-day residual effects that are dose-dependent and resolve over time, which is consistent with this study, where sunobinop 6 mg resulted in greater next-day residual effects, while sunobinop doses of 2 mg or less produced limited next-day residual effects.

Consistent with previous sunobinop studies, (Cipriano et al., 2024; Whiteside et al., 2024), somnolence was the most commonly reported AE in this study, and sunobinop was safe and generally well tolerated. In addition, this study helped define the next-day residual effects of sunobinop across a range of doses from 0.2 mg to 6 mg, with minimal next-day effects observed at doses of 2 mg and below. As such, data from this study can assist with dose selection in future studies of sunobinop.

The limitations of this study include the small sample size, a single treatment administration, and the study of healthy participants. Future studies should address these limitations.

## Conclusion

Next-day residual effects following sunobinop administration were dose-dependent. There were no statistically significant differences on any of the PD endpoints measured at sunobinop doses <2 mg. At 2 mg, PD effects were small and variable. The last significant differences between sunobinop 2 mg and placebo on the DSST, KSS, and body sway were observed at 12 h, 16.5 h, and 13.5 h postdose, respectively. Sunobinop 6 mg resulted in a large and consistent effect on PD at all timepoints up to 16.5–18 h postdose, and all PD effects resolved by 22 h postdose.

Overall, a nighttime oral dose of sunobinop up to 2 mg was safe and generally well tolerated in healthy participants with limited next-day residual effects that were consistent with other sedative/hypnotic drugs. These results defined the next-day residual effects of sunobinop across a broad range of doses and support its continued clinical development.

## Data Availability

Raw data supporting the conclusion of this article will be made available upon request to Garth Whiteside at garth.whiteside@imbriumthera.com.
